# The long noncoding RNA family gets bigger: thousands of new loci identified in natural *Arabidopsis* accessions

**DOI:** 10.1093/plcell/koad242

**Published:** 2023-09-19

**Authors:** Johan Zicola

**Affiliations:** Assistant Features Editor, The Plant Cell, American Society of Plant Biologists; Department of Crop Sciences and Center for Integrated Breeding Research, The University of Göttingen, Göttingen, Germany

Long noncoding RNAs (lncRNAs) are a class of RNA molecules that are longer than 200 nucleotides and are not translated into proteins. lncRNAs play important regulatory roles in various cellular processes, such as gene expression and epigenetic regulation. They contribute to the complexity of gene regulation and are involved in shaping an organism's development, function, and response to its environment (Mattick et al. 2023). In plants, the regulatory functions of about 24 lncRNAs have been elucidated (Palos et al. 2023), but there is little doubt that these cases only represent the tip of the iceberg.

In this issue of *The Plant Cell*, **Aleksandra Kornienko and coauthors** (Kornienko et al. 2023) identified about 11,300 lncRNA loci using transcriptomic data (RNA sequencing) of 5 different tissues/developmental stages and 499 accessions. About 72% of these lncRNAs are antisense to protein-coding genes (AS lncRNAs), 20% are located in intergenic regions (lincRNAs), and 6% are antisense to transposable element (TE) genes. The number of lncRNA loci found exceeds by 6-fold the Araport11 annotation based on the reference accession Col-0. This increase in number is due to the high variation in lncRNA expression, with most loci being either expressed in fewer than 5% of all accessions or in a tissue-specific manner (see Fig.). These results indicate that the expression of most lncRNAs loci is accession and tissue specific, with the basal state being a silenced one.

**Figure.**
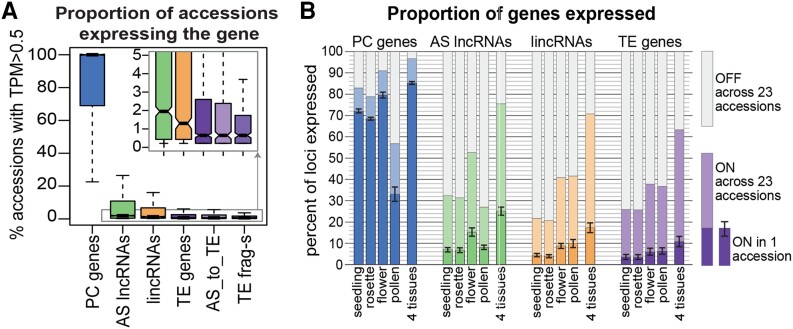
Variability in expression of lncRNAs across accessions and tissues. **A)** Proportion of accessions (n = 461) expressing the different types of lncRNAs. **B)** Proportion of expressed lncRNA loci across 4 tissues in 23 accessions. AS, antisense; linc, long intergenic noncoding; PC, protein-coding, TE, transposable element; TPM, transcripts per million mapped reads.

Since most lncRNAs are silenced in any given accession, the authors investigated the epigenetic marks involved in silencing. They distinguished the silencing pathways for protein-coding genes and TEs using specific histone marks and found that the epigenetic features of antisense lncRNAs were close to protein-coding genes, while lincRNAs, which by definition do not overlap genes, showed epigenetic patterns intermediate between TEs and protein-coding genes. They also found a clear bimodal distribution of CG DNA methylation at lincRNAs, where high DNA methylation levels were associated with low lincRNA expression. By comparing lncRNA expression and DNA methylation levels across 444 accessions, they could explain 50% and 20% of expression variation for lincRNAs and AS lncRNAs, respectively. These results suggest that lincRNA expression is partly controlled by the TE silencing pathway. Therefore, the authors decided to focus on lincRNAs and their similarities with TEs.

About 50% of lincRNAs were found to contain known TE sequences, and these lincRNAs were enriched in epigenetic marks typical of TEs and are less likely to be expressed than lincRNAs with no TE sequences. The copy number of lincRNAs also affects their expression variability and epigenetic profile, with high-copy lincRNAs more likely to be silenced and to contain TE sequences. At first sight, these results could suggest that TE-containing lincRNAs are simply unannotated TEs. However, the authors argue against this hypothesis by showing that TE pieces contained within a given lincRNA are much shorter in size than typical TEs and often come from different TE families. However, the distinction between lncRNAs and TEs is not always obvious and their origins are most likely intertwined (Lee et al. 2019).

In addition to lincRNAs displaying a TE-like epigenetic silencing profile, they also found a fraction of lincRNAs displaying an enrichment in trimethylation of lysine 27 histone 3 (H3K27me3), a hallmark of protein-coding gene silencing by the Polycomb repressive complex 2. These lincRNAs were usually without TE sequences and mostly present in 1 copy, making them look like PC genes.

Overall, the authors showed that many lncRNAs could be identified by using a large panel of *Arabidopsis* accessions and different tissue types. They showed that some of the lncRNAs located in intergenic regions can display TE-like silencing via dimethylation of lysine 9 histone 3 (H3K9me2) and DNA methylation, and protein-coding gene-like silencing via H3K27me3. Half of the lincRNAs contain small TE sequences, opening new questions on the origins of these mosaic sequences and their regulatory functions. The resource provided here will facilitate future research on the noncoding “dark matter” of the *Arabidopsis* genome.

## References

[koad242-B1] Kornienko AE , Nizhynska V, Morales AM, Pisupati R, Nordborg M. Population-level annotation of lncRNAs in Arabidopsis reveals extensive expression variation associated with transposable element–like silencing. Plant Cell. 2023:36 (1):85–111. 10.1093/plcell/koad233 37683092 PMC10734619

[koad242-B2] Lee H , Zhang Z, Krause HM. Long noncoding RNAs and repetitive elements: junk or intimate evolutionary partners? Trends Genet. 2019:35(12):892–902. 10.1016/j.tig.2019.09.006 31662190

[koad242-B3] Mattick JS , Amaral PP, Carninci P, Carpenter S, Chang HY, Chen LL, Chen R, Dean C, Dinger ME, Fitzgerald KA, et al Long non-coding RNAs: definitions, functions, challenges and recommendations. Nat Rev Mol Cell Biol. 2023:24(6):430–447. 10.1038/s41580-022-00566-8 36596869 PMC10213152

[koad242-B4] Palos K , Yu L, Railey CE, Nelson Dittrich AC, Nelson ADL. Linking discoveries, mechanisms, and technologies to develop a clearer perspective on plant long noncoding RNAs. Plant Cell. 2023:35(6):1762–1786. 10.1093/plcell/koad027 36738093 PMC10226578

